# The potential for genomics to help guide preventive strategies in psychiatry

**DOI:** 10.1001/jamapsychiatry.2026.2583

**Published:** 2026-11-01

**Authors:** Katherine L. Musliner, Eva C. Schulte, Gerome Breen, Giuseppe Fanelli, Andreas J. Forstner, Philip R. Jansen, Kimberley M. Kendall, Jurjen Luykx, Vincent Millischer, Sergi Papiol, Evangelos Vassos, Boris Chaumette, Wouter J. Peyrot

**Affiliations:** 1 Department of Affective Disorders, Aarhus University Hospital, Aarhus, Denmark; 2 Department of Clinical Medicine, Aarhus University, Aarhus, Denmark; 3 University of Bonn, University Hospital Bonn, Department of Psychiatry & Psychotherapy, Bonn, Germany; 4 University of Bonn, University Hospital Bonn, Institute of Human Genetics, Bonn, Germany; 5 Institute of Psychiatric Phenomics and Genomics (IPPG), University Hospital, LMU Munich, Munich, Germany; 6 German Center for Mental Health (DZPG), partner site Munich-Augsburg; 7 Social, Genetic & Developmental Psychiatry Centre, Institute of Psychiatry, Psychology and Neuroscience, King’s College London, London, UK; 8 National Institute for Health and Care Research (NIHR) Maudsley Biomedical Research Centre at South London and Maudsley NHS Foundation Trust and King’s College London, London, UK; 9 Department of Medicine, University of Udine, Udine, Italy; 10 Department of Human Genetics, Radboud University Medical Center, Nijmegen, The Netherlands; 11 Institute of Neuroscience and Medicine (INM-1), Research Center Jülich, Jülich, Germany; 12 Amsterdam UMC, Department of Human Genetics, University of Amsterdam, Amsterdam, Netherlands; 13 Emma Center for Personalized Medicine, Amsterdam UMC, Amsterdam, the Netherlands; 14 Centre for Neuropsychiatric Genetics and Genomics, Cardiff University, Cardiff, UK; 15 Department of Psychiatry, Amsterdam University Medical Center, Amsterdam, the Netherlands; 16 Amsterdam Neuroscience and Public Health Research Institutes, Amsterdam, The Netherlands; 17 GGZ inGeest Mental Health Care, Amsterdam, The Netherlands; 18 Department of Psychiatry and Neuropsychology, School for Mental Health and Neuroscience, Maastricht University Medical Center, Maastricht, the Netherlands; 19 Department of Psychiatry and Psychotherapy, Clinical Division of General Psychiatry, Medical University of Vienna, Vienna, Austria; 20 Comprehensive Center for Clinical Neurosciences and Mental Health, Medical University of Vienna, Vienna, Austria; 21 Department of Molecular Medicine and Surgery, Karolinska Institutet, Stockholm, Sweden; 22 Center for Molecular Medicine, Karolinska University Hospital, Stockholm, Sweden; 23 Max Planck Institute of Psychiatry, Munich, Germany; 24 Université Paris Cité, Institute of Psychiatry and Neuroscience of Paris ( INSERM U1266), Institut Pasteur ( CNRS UMR3571), GHU Paris Psychiatrie et Neurociences, Paris, France; 25 Department of Psychiatry, McGill University, Montreal, Canada; 26 Department of Psychiatry, Amsterdam UMC, Vrije Universiteit, Amsterdam, The Netherlands; 27 Amsterdam Public Health, Amsterdam, The Netherlands

## Abstract

**Importance:**

Psychiatric disorders are a major source of disability and premature mortality worldwide. Advances in genomics have clarified parts of their genetic architecture, prompting questions about whether genomic information can inform preventive strategies for psychiatric disorders in clinical practice. In this Perspective, we describe potential benefits and harms of using genomic information – particularly polygenic scores (PGS) and rare variants – to guide prevention in psychiatry.

**Observations:**

Broad application of genomic testing in universal or selective primary prevention is not currently supported by evidence of net clinical benefit, given modest absolute risk differences, limited individual-level predictive performance, and the potential for psychological harm. The possible value of genomic testing increases for indicated primary prevention, as prior probability rises and diagnostic uncertainty becomes clinically relevant. The most compelling opportunity lies in early care after individuals enter the psychiatric treatment system but before diagnostic trajectories fully crystallize. In this “zone of diagnostic uncertainty,” testing for rare, high-impact variants can shorten diagnostic delays, inform prognosis, and guide monitoring and treatment, provided results are communicated appropriately. PGS may contribute to risk stratification when applied in high-risk clinical populations and integrated with non-genetic factors. In secondary and tertiary prevention, pharmacogenomics provides actionable benefits for medication choice and safety, and rare variant testing has clear value in selected instances. Beyond psychiatric outcomes, genomic testing could potentially help address the profound mortality gap in severe mental illness by identifying elevated risk for somatic comorbidities, thus supporting more proactive, integrated care.

**Conclusions and relevance:**

Currently, genomic testing in psychiatry should not be used for population-wide screening but may have value as a targeted clinical resource whose utility depends on timing, context, and purpose. When applied judiciously and embedded within comprehensive, ethically grounded care pathways, genomic testing holds promise as one component of a more precise, preventive, and patient-centered psychiatry.

## Background

Psychiatric disorders are common and burdensome, with substantial global impacts on morbidity and mortality.^1^ Social and environmental factors are known to play a major role in the onset and course of psychiatric disorders^2,3^, yet the role of genetic liability is also substantial and spans diagnostic categories.^4^ Over the past two decades, considerable advances in genotyping and sequencing technologies together with sustained global investment and large-scale international collaborations have transformed the evidence base on genomic contributions to psychiatric disorders. After decades of substantial public investment and methodological progress, advancing from gene discovery to rigorous assessment of the potential for clinical translation represents a necessary next step for psychiatric genomics. In this paper, we approach the question of the potential use of genomics in psychiatry from a preventive medicine perspective, evaluating the arguments for and against its potential utility within different preventive intervention contexts. Rather than asking whether genetic testing can improve diagnosis or risk prediction in principle, we evaluate whether genomic information can support prevention in ways that are clinically actionable, ethically acceptable, and likely to yield net benefits.

### Genetic architecture of psychiatric disorders

Over the past two decades, new technologies such as cost effective high-throughput genotyping microarrays, next-generation sequencing and bioinformatics pipelines have facilitated the study of genetic variation across the genome, at scale. In psychiatry, this enabled large international collaborations, such as the Psychiatric Genomics Consortium^5^, to achieve the sample sizes needed to detect variants across the full range of effect sizes. Consequently, our understanding of the genetic architecture of psychiatric disorders is more complete than ever. Both GWAS and whole genome sequencing (WGS) studies indicate that psychiatric disorders are highly polygenic, meaning that genetic risk is attributable to small, primarily additive effects of thousands of common variants (>1% frequency).^6^ In some cases, disorders are linked to rare variants, including copy number variants and rare coding variants^7–11^. Rare variants have been identified in 30-50% of patients with intellectual disability (ID)^12,13^, 14% of patients with autism spectrum disorder (ASD),^14^ 13% of patients with attention deficit hyperactivity disorder (ADHD)^15^ and 6% of patients with schizophrenia.^16^ Methods have been developed to quantify polygenic liability into scores summarizing an individual’s burden of common risk alleles.^17^ These polygenic scores (PGS) are currently the primary common-variant approach for potential clinical application, due to their conceptual simplicity and scalability.^18^

### Preventive interventions – a framework

**Figure 1:**
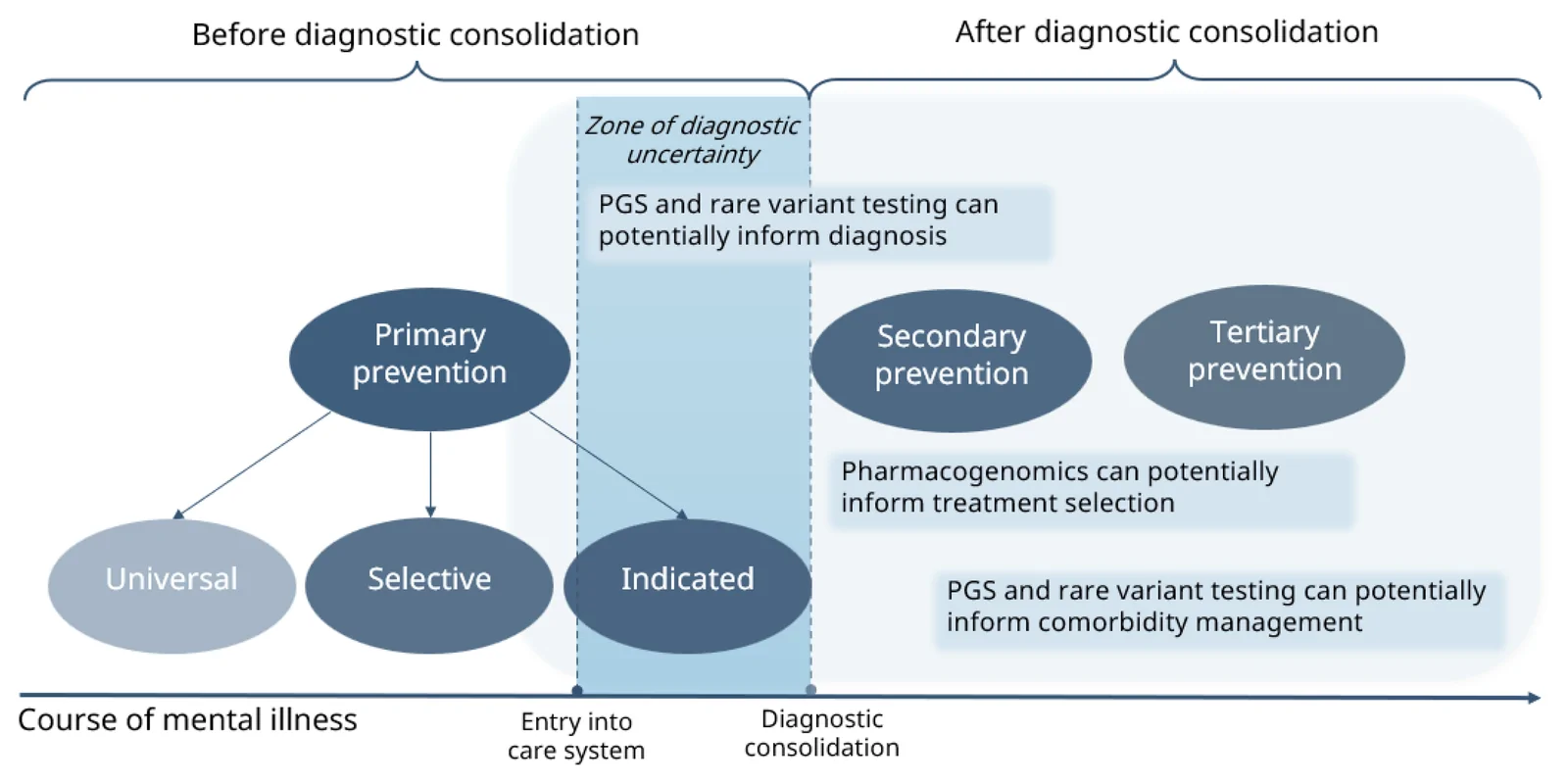
Potential for genomic testing to inform prevention in psychiatry *Note*. This figure illustrates the hypothesized timing and potential clinical utility of genomic information across the course of psychiatric illness, from the preclinical period through diagnostic consolidation and subsequent clinical management. Prior to diagnostic consolidation, genomic information—including polygenic scores (PGS) and rare variant testing—may potentially inform diagnostic clarification, particularly around entry into the care system. After diagnostic consolidation, different forms of genomic information may have distinct clinical applications: pharmacogenomic data may potentially inform treatment selection during secondary prevention, whereas PGS and rare variant testing may potentially inform comorbidity risk assessment and management during secondary and tertiary prevention. Prevention categories (universal, selective, indicated, primary, secondary, and tertiary) are shown schematically to reflect relative timing rather than discrete clinical stages. The timing and clinical relevance of genomic testing are illustrative and may vary by disorder, clinical context, and health care setting.

Preventive interventions are typically conceptualized using two complementary frameworks. The first, introduced by Leavell and Clark in 1958, categorizes prevention into primary, secondary and tertiary.^19^ Primary prevention (e.g. vaccines) aims to prevent disease onset. Secondary prevention (e.g. Pap smears) aims to halt or reverse disease progression through early identification. Tertiary prevention (e.g. occupational therapy after stroke) aims to reduce disability and complications, improve rehabilitation and prevent relapses once a disease is established. The second framework, introduced by Gorden in 1983, categorizes primary prevention into universal, selective and indicated strategies.^20^ Universal interventions (e.g. water fluoridation) target the entire population. Selective interventions (e.g. prophylactic mastectomy in carriers of pathogenic BRCA variants) target individuals at high risk based on group membership. Indicated interventions (e.g. lifestyle counseling for pre-hypertension) target individuals at high risk based on individual characteristics. In 2004, the World Health Organization (WHO) adopted a hybrid of the two in which primary prevention is subdivided into the categories of universal, selective and indicated (Figure 1).^21^ This hybrid framework has the benefit of holism; however, in psychiatry, where biological markers of disease onset are lacking, the boundary between indicated primary prevention and secondary prevention can become murky.

Proposed prevention strategies for psychiatric disorders include universal interventions such as school-based programs, selective interventions such as psychoeducation for children of people with psychiatric disorders, and indicated interventions such as cognitive behavioral therapy (CBT) for individuals with subclinical psychiatric symptoms.^22,23^ Much research has focused on preventing progression to schizophrenia in individuals at clinical high risk for psychosis (CHR-P)^24^, although this approach is not free of criticism.^25^ Overall, the evidence for the effectiveness of primary prevention interventions in psychiatry is limited^22–24^.

### Universal primary prevention

A classic example of universal primary prevention using genetic testing is newborn screening for rare, treatable genetic disorders, some of which can present with psychiatric symptoms.^26^ Existing panels already include conditions such as X-linked adrenoleukodystrophy in the United States^27^ and Wilson’s disease and homocystinuria in parts of Central America, North Africa and the Middle East.^28^ WGS has recently been proposed for newborn screening, but its application is currently limited primarily to neurometabolic disorders.^29,30^ Rare, high impact variants associated with psychiatric disorders such as ASD,^9^ schizophrenia^8^, and ADHD,^11^ are individually uncommon but collectively present in a meaningful proportion of cases^14,16^. However, these variants typically show variable penetrance and considerable phenotypic heterogeneity.^31^ For example, the 22q11.2 deletion—the most studied rare variant associated with schizophrenia—accounts for only 0.5-1% of cases, and 75% of carriers never develop schizophrenia.^32^ More importantly, these variants are arguably not sufficiently clinically actionable within a universal primary prevention setting, as there are currently no evidence-based interventions shown to prevent or mitigate future psychiatric symptoms in asymptomatic carriers of rare variants. Although some supportive or low-risk interventions may ultimately prove beneficial, their effectiveness remains uncertain and unintended consequences including psychological distress and stigma must also be considered. Given the absence of proven preventive interventions and the uncertain balance of benefits and harms, our view is that screening for these variants in the context of universal primary prevention in psychiatry is not advisable. However, the balance of these considerations may change in the future if further evidence supporting effective preventive measures emerges.

GWAS consistently demonstrate differences in PGS distributions between cases and controls^33–35^, and population-based studies show that PGS prospectively predict later psychiatric disorders^36–38^. However, absolute risk differences in the general population are small, even at the extremes of PGS distributions^36^. This reflects limitations imposed by prior probability: common disorders such as major depressive disorder (MDD) are prevalent within the population but only modestly heritable (30-40%)^39^, yielding modest absolute risk differences. In the Danish iPSYCH register-based case-cohort study – to our knowledge the only source to date of population-based absolute risk estimates for psychiatric disorders stratified by PGS – risk for secondary care-treated MDD was 3% in the lowest PGS quintile (i.e. bottom 20%) vs. 8% in the highest.^38^ Although disorders such as schizophrenia, ASD and ADHD have higher heritability (60-90%), their lower population prevalence similarly constrains absolute risk (e.g., in iPSYCH, 1.6% vs. 2.3% for ASD; 1.4% vs. 3.3% for schizophrenia)^38^. Incorporating rare CNVs modestly improves prediction, yet even individuals in the highest risk groups reach only 5-10% absolute risk for diagnosis by age 35.^38^ While such risk levels may justify intervention in selected clinical contexts, their use in universal primary prevention raises concern. Experimental studies show that disclosure of elevated genetic risk for depression increases recall of previous depressive symptoms^40^ and reduces perceived coping capacity^41^. Genomic risk can be difficult to communicate^42^, and sharing this information could potentially promote biological determinism and psychological harm, although prospective data on downstream clinical effects are lacking. Moreover, while lifestyle interventions such as exercise or diet improvement may reduce risk at the population level^43^, evidence that any intervention can reliably prevent psychiatric disorders at an individual level remains limited, with no clear indication that such effects are modified by genetic background. Taken together, current evidence suggests that the potential harm of genomic testing in universal psychiatric prevention outweighs its benefits.

### Selective primary prevention

Many population subgroups have elevated risk for psychiatric disorders, including military veterans, individuals with a history of migration or exposure to childhood trauma or poverty^2,3,44^. However, even within these groups, absolute risk gains through PGS stratification generally remain low^36,45^ (e.g. 0.9% in the lowest vs. 4.0% in the highest PGS decile for schizophrenia in European-ancestry individuals from the Million Veterans Program)^46^, and the potential for stigma or psychological harm persists; thus, the risk/benefit considerations of genomic testing are similar to those in universal primary prevention. An important exception is children of individuals with severe psychiatric disorders, approximately 55% of whom will develop a psychiatric disorder themselves^47^ and for whom genomic testing may refine estimates of individual risk beyond family history alone^48,49^. For these high-risk individuals, not only is the prior probability of psychiatric disorders substantially elevated, but the potential psychological harms of disclosing genetic risk information are likely attenuated, as awareness of elevated familial risk—and associated distress regarding personal or offspring risk—is often already present. In this context, genomic testing may either provide reassurance when risk is lower than anticipated or help clarify risk and guide next steps when it is higher. Accordingly, the balance of risks and benefits may favor consideration of genomic testing in this group, although clinical trial data confirming these benefits is still needed.

Another potential exception may be the use of PGS for risk stratification in carriers of rare genetic variants, another subgroup where the prior probability of mental illness is substantially increased. Emerging evidence suggests that common and rare genetic variation act jointly to influence psychiatric outcomes, with polygenic background contributing to the incomplete penetrance and phenotypic heterogeneity observed among carriers of rare variants. For example, among individuals with the 22q11.2 deletion, higher PGS for schizophrenia have been associated with an increased likelihood of developing psychosis compared with unaffected carriers^50,51^ However, uncertainty remains regarding the predictive value of PGS in this subgroup, and additional evidence of net benefit is needed before this approach can be recommended for routine implementation.

### Indicated primary prevention

While genomic testing appears poorly suited for universal and most selective primary prevention in psychiatry, its potential value may emerge in the context of indicated primary prevention. Specifically, we see the largest potential benefit of genomic testing in psychiatry within the period after an individual has come into contact with mental health care services but before symptoms have coalesced into a stable diagnosis, a process that may take years and involve multiple diagnostic labels^52^. Within this “zone of diagnostic uncertainty” (Figure 1), screening for rare, large-effect genetic variants may be warranted for some individuals, particularly those with other characteristics such as comorbid intellectual disability^16^, extreme early onset, cognitive impairment, neurological symptoms, dysmorphic features or congenital malformations.^53,54^ Accurate genetic diagnosis at this stage can guide monitoring for psychiatric and medical comorbidities, inform treatment choices (including medication selection) and identify at-risk family members.^55^ Importantly, it has also been shown to be generally acceptable to patients.^56^

PGS could also potentially be useful for refining personalized risk at this stage, potentially improving early diagnosis and/or diagnostic refinement^57–60^. The key is to apply PGS within groups where the prior probability of disorder is sufficiently elevated. For example, individuals with CHR-P, where risk for schizophrenia is around 25%, as opposed to the general population where the prevalence of schizophrenia is only 1%.^61^ Within such groups, PGS could help stratify individuals into risk groups ranging from e.g. 5-10% in the lowest decile-strata to 50-60% in the highest decile^59^. We believe such stratification yields appreciable potential to help in guiding preventive interventions. However, it is important to consider the reference group. If the criteria used to define eligibility for indicated prevention are associated with increased polygenic liability, there may be insufficient variation left for effective risk stratification^62^. Evaluating the potential utility of PGS-based risk stratification in differentially defined indicated populations represents an important area for future research.

### Secondary and tertiary prevention

Once signs and symptoms have coalesced into a clear clinical presentation, genomic testing may have a role in secondary and tertiary prevention. The broadest and most immediate potential application is pharmacogenetics^63–65^, which could improve on the trial-and-error approach that currently characterizes much psychotropic prescribing and that contributes to high treatment discontinuation and suboptimal outcomes.^66,67^ Genetic variation in drug-metabolizing enzymes such as CYP2D6 and CYP2C19 has a well-established and often clinically meaningful impact on pharmacokinetic parameters (e.g., drug plasma levels), with differences in drug exposure of up to several-fold across metabolizer groups.^68^ However, translation to downstream clinical outcomes such as symptom improvement or remission is modest and variable. Meta-analytic evidence in MDD suggests that pharmacogenomic-guided treatment is associated with improved outcomes compared with treatment as usual (e.g., ORs ∼1.4 for remission), indicating a modest but clinically relevant benefit.^69^ Testing for genetic variants associated with severe adverse drug reactions is recommended in specific contexts and represents a clear and actionable application of pharmacogenetics in clinical practice (i.e. HLA genotyping before prescription of carbamazepine).^68^ Nonetheless, the proportion of variance explained in clinical outcomes remains limited, reflecting the multifactorial nature of treatment response and the contribution of non-genetic factors.

**Figure 2:**
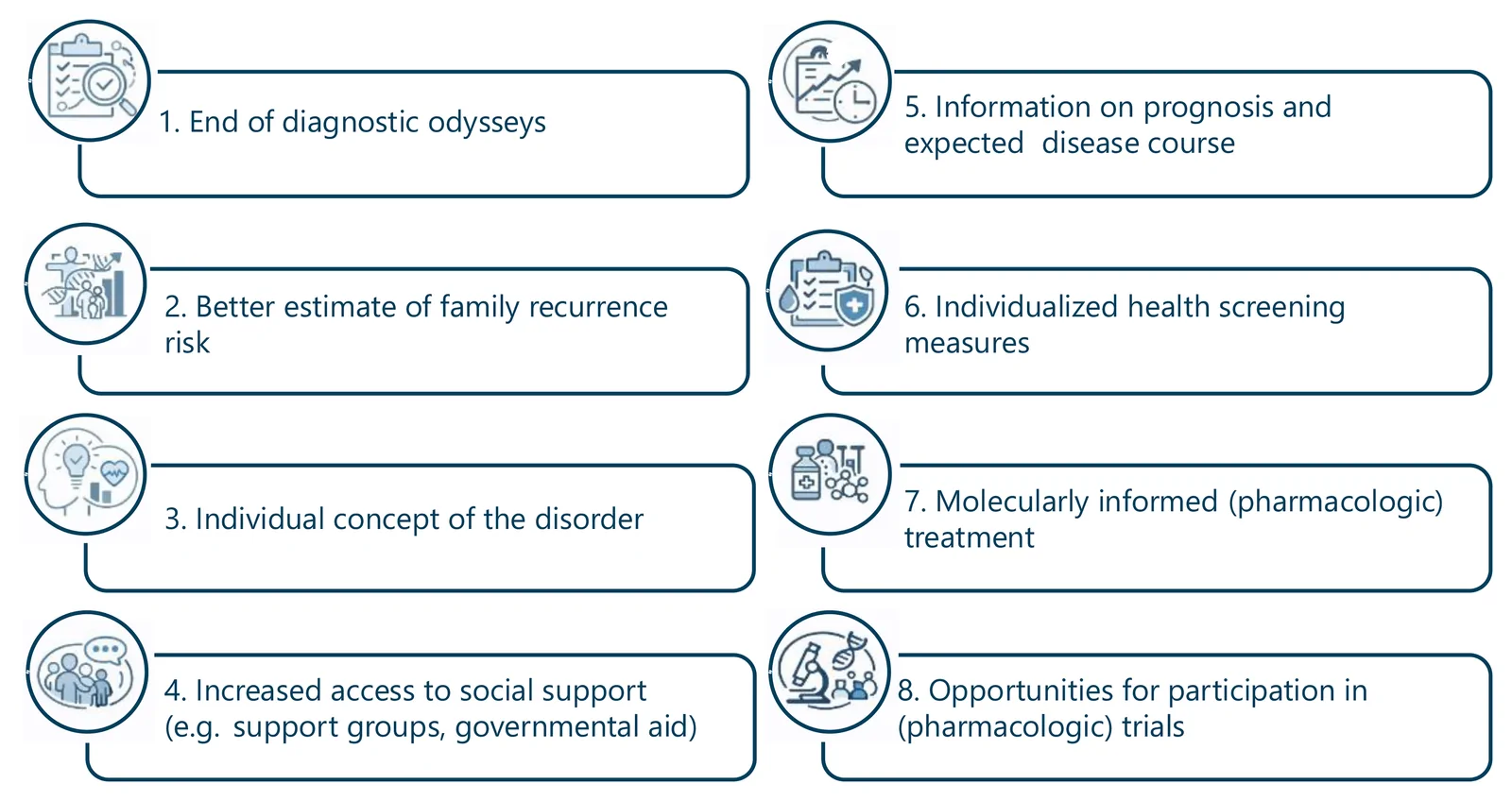
Potential benefits of genetic testing for patients with psychiatric disorders *Note*. (1) molecular diagnoses can *end diagnostic odysseys* at any time during the course of disease and provide (2) information on family *recurrence risks*; (3) they can impact an individual’s and a family’s *concept of the disorder* with potential for a reduction in stigma^42,43^ as well as improved treatment adherence; (4) lead to *increased social support* in the form of family support groups for specific disorders or governmental aid, likely improving the overall management of the disorder^44^ and thereby, potentially, the functional outcome; (5) more concrete information on *prognosis and expected disease course* can be preventive in, for example, counteracting or providing early treatment of (mostly somatic) disease manifestations like, for instance, orthopedic manifestations and by leading to the (6) implementation of *syndrome-specific screening measures* as already depicted above; an identified rare genetic cause of a psychiatric disorder can also lead to better (7) *informed (pharmacologic) treatment* ^31^ and, as clinical trials for rare genetic conditions are increasingly implemented, (8) *enable participation* in such *trials*, permitting precise therapeutic interventions.

Testing for rare genetic disorders may also yield benefits at later stages of illness (Figure 2). In some instances, features only apparent after the ultimate psychiatric diagnosis, such as treatment resistance, may themselves signal an underlying rare genetic disorder. One hallmark study found that up to 48.2% of individuals with ‘severe, extremely treatment-resistant schizophrenia’ had damaging missense or loss-of-function variants in schizophrenia-associated genes^70^ and rare copy number variants (>30kb) have been linked to worse response to antipsychotic medications^71^. This suggests that drug resistance may warrant consideration of diagnostic genetic testing, although this approach has not yet been evaluated in large prospective studies. Testing in both children and adults with qualifying ID has already made its way into several expert opinions^72^ and national treatment recommendations.^53,73^ An accurate molecular diagnosis at this stage can inform treatment selection^55^ and clarify prognosis and disease course, including the risk of somatic comorbidities, thereby enabling earlier recognition and management. More broadly, it can end prolonged diagnostic odysseys, providing explanation and relief to patients and their families. Genetic diagnosis can also improve treatment adherence—for example by facilitating earlier identification of the optimal treatment approach and thus increasing treatment efficacy— reduce stigma,^74,75^ and increase access to family or governmental support, with potential downstream benefits for functional outcomes.^76^ Finally, although not directly related to secondary or tertiary prevention, identification of a rare genetic disorder can inform recurrence risk for family planning and facilitate participation in emerging clinical trials.

Whereas testing for rare, large-effect variants yields large potential value for secondary and tertiary prevention in some, the evidence supporting clinical use of psychiatric PGS at this stage is unclear. Several studies have shown that higher polygenic liability for schizophrenia is associated with poorer treatment outcomes across disorders,^63,77,78^ and psychiatric PGS show modest associations with antidepressant treatment response in MDD,^79^ diminished lithium efficacy in bipolar disorder^80–82^, and greater likelihood of clozapine use and treatment resistance in patients with schizophrenia^83^. However, the associations are modest and the proportion of variance explained by PGS is small.^63^ Studies consistently find small associations between PGS and course trajectories and severity in MDD,^84,85^ ADHD,^86^ and bipolar disorder^87^. but PGS stratification yielded only small differences in absolute risk for recurrence in MDD^88^ and incorporating PGS into prediction models for first-episode psychosis or bipolar disorder did not meaningfully improve their performance for a range of clinical outcomes^89,90^. This pattern may indicate that PGS are better suited to predicting disorder onset, diagnostic transition or progression—what we define here as indicated primary prevention—than to predicting illness course or treatment response. Alternatively, it may reflect limitations of current PGS, which are largely trained on case-control phenotypes capturing disorder susceptibility rather than longitudinal course or treatment outcomes. If the genetic architecture of prognosis differs from that of disorder risk, existing psychiatric PGS would be expected to show limited utility for secondary or tertiary prevention.

PGS for physical health traits such as cardiovascular disease or obesity may have greater clinical relevance in psychiatric populations for predicting somatic comorbidities or medication-related side effects. Individuals with psychiatric disorders experience substantially reduced life expectancy^91–94^ due largely to higher rates of physical illness. Genetic risk stratification could help address this disparity by identifying patients at elevated risk for somatic disease. Consistent with this, recent analyses in UK cohorts found that polygenic liability for physical health conditions was associated with physical health comorbidities among individuals with severe mental illness, suggesting a potential role for PGS in this context.^95^

### Next steps and considerations

Moving forward, efforts should focus on implementing diagnostic genetic testing for rare variants in individuals for whom results are most likely to inform clinical management, while also increasing the accessibility of these services. Although guidelines in many countries recommend genetic testing in individuals with ASD, the SPARK study found that of 21,532 individuals with ASD, only 6.8% reported having been tested by chromosomal microarray and only 1% had received whole exome sequencing, highlighting a large existing diagnostic gap^96^. Delays in diagnosing rare neuropsychiatric genetic disorders average more than seven years^55^ and could potentially be reduced through broader testing in indicated populations.

With regards to PGS, there remain substantial methodological limitations in predictive capacity and stability which must be overcome before PGS are ready for use in clinical practice. Current PGS also face substantial cross-ancestry transportability issues due to the predominance of European-ancestry samples in GWAS which impacts allele frequencies, LD structure and calibration^97–99^. Genetic ancestry can also affect the interpretation of rare variants, although through different mechanisms. Rare variant frequencies and background variation differ across ancestral populations, and reference databases remain comparatively sparce for individuals of non-European ancestry, complicating accurate variant interpretation. This underscores the value of WGS, which can help clarify how genetic variants influence disease risk across diverse ancestral groups. Accurate assignment of polygenic load to the appropriate population-level risk strata requires both a thorough understanding of an individual’s genetic ancestry and access to suitable ancestry-specific reference panels, which are currently available in some countries (e.g. Denmark^100^) but not others. Clinical trials are needed to prospectively evaluate whether refining risk estimates using polygenic stratification leads to more tailored prevention, and whether this in turn leads to improved outcomes without undue harm. Clinical implementation of both common and rare variants would likely benefit greatly from integration with each other^101,102^, and with non-genetic risk factors including clinical history, environmental exposures, and social determinants. They may also ultimately benefit from incorporation of other biological layers such as transcriptomic, proteomic, or metabolomic measures in multimodal models.

Greater use of genetic testing in psychiatry comes with additional financial costs, heavier workload for laboratory specialists and the need to train more genetic counselors. WGS also invariably leads to the added burden of identifying variants of unknown significance, which can be challenging for clinicians to interpret and their meaning difficult to communicate to patients due to their ambiguous nature. Finally, ethical issues must be thoroughly considered.^103^ Genetic information in psychiatry carries a heightened risk of misinterpretation, stigma, and psychological burden, particularly when communicated outside structured care pathways. While psychiatric genetics seems to be largely acceptable to patients and their families,^56^ their engagement is necessary to ensure that implementation strategies reflect patient priorities and promote trust.

Finally, we emphasize the importance of careful and empowering risk communication. Genetic risk is often perceived as deterministic because the genetic contribution to risk cannot be changed. This misconception, and others, should be carefully addressed. Although genetic risk itself cannot be modified, patients can be empowered by focusing on aspects they can control, such as avoiding known risk factors like cannabis use, maintaining a healthy lifestyle, and adhering to treatment. The ‘mental jar’ model developed by Jehannine Austin provides a powerful and evidence-based framework for this type of risk communication. Psychiatric genetic counseling using this approach has been shown to increase patient empowerment, reduce internalized stigma, and decrease psychiatric symptoms over time^104–106^.

## Conclusion

In sum, we argue that genomic testing in psychiatry should not be seen as a population-wide screening tool but as a targeted clinical resource whose value depends on time, context, and purpose. The most promising window lies in indicated primary prevention and early stages of care, after individuals have entered the mental health system but before diagnostic trajectories have fully crystallized. Methodological, ethical, and implementation challenges remain, and prospective clinical trials are essential to determine whether genetically informed care improves outcomes. When applied judiciously and embedded within comprehensive, ethically grounded care pathways, genomic testing may be a useful component of a more precise, preventive, and patient-centered psychiatry.
